# Language switching training modulates the neural network of non-linguistic cognitive control

**DOI:** 10.1371/journal.pone.0247100

**Published:** 2021-04-15

**Authors:** Mo Chen, Fengyang Ma, Zhaoqi Zhang, Shuhua Li, Man Zhang, Qiming Yuan, Junjie Wu, Chunming Lu, Taomei Guo

**Affiliations:** 1 State Key Laboratory of Cognitive Neuroscience and Learning & IDG/McGovern Institute for Brain Research, Beijing Normal University, Beijing, P. R. China; 2 School of Education, University of Cincinnati, Cincinnati, Ohio, United States of America; 3 Key Research Base of Humanities and Social Sciences of the Ministry of Education, Academy of Psychology and Behavior, Tianjin Normal University, Tianjin, China; 4 Center for Collaboration and Innovation in Brain and Learning Sciences, Beijing Normal University, Beijing, P. R. China; University of California, San Francisco, UNITED STATES

## Abstract

Bilingual language experience, such as switching between languages, has been shown to shape both cognitive and neural mechanisms of non-linguistic cognitive control. However, the neural adaptations induced by language switching remain unclear. Using fMRI, the current study examined the impact of short-term language switching training on the neural network of domain-general cognitive control for unbalanced Chinese-English bilinguals. Effective connectivity maps were constructed by using the extended unified structural equation models (euSEM) within 10 common brain regions involved in both language control and domain-general cognitive control. Results showed that, the dorsal anterior cingulate cortex/pre-supplementary motor area (dACC/pre-SMA) lost connection from the right thalamus after training, suggesting that less neural connectivity was required to complete the same domain-general cognitive control task. These findings not only provide direct evidence for the modulation of language switching training on the neural interaction of domain-general cognitive control, but also have important implications for revealing the potential neurocognitive adaptation effects of specific bilingual language experiences.

## Introduction

With economic globalization, an increasing number of people use two or more languages in their daily lives. These individuals are termed bilinguals or multilinguals. Different from monolinguals who can speak only one language, bilinguals need to switch between languages. In the bilingual literature, it has been hypothesized that in these processes, executive control is recruited to coordinate their two languages [1]. Therefore, long-term bilingual experience could potentially modulate domain-general executive control functions to a certain degree [2]. In the current study, we aim to investigate how language switching, one important aspect of bilingual experiences, impacts the cognitive control neural networks.

In the investigation of bilingualism, one fundamental and hotly debated issue is to determine its consequences on the brain and mind [3, 4]. For example, many studies have examined the influence of bilingual experience on bilingual executive control, but reported mixed evidence. One line of research has compared bilinguals with their monolingual peers, reporting that when completing non-linguistic executive function tasks, particularly tasks engaging conflict control functions, e.g., the Simon Task, the Flanker Task, and the Stroop Task, bilinguals exhibit a smaller conflict effect (i.e., the difference between inconsistent and consistent conditions), suggesting cognitive accommodations to bilingual experience in cognitive control [2, 5, 6]. Furthermore, a number of functional magnetic resonance imaging (fMRI) and magneto-encephalography (MEG) studies have explored how bilingual language experience modulates brain plasticity by comparing bilinguals and monolinguals in the neural mechanisms of executive control [7–12]. So far, brain imaging evidence available has shown that activation patterns in a number of brain regions differ between bilinguals and monolinguals when they perform cognitive control tasks [7, 8, 11, 13, 14]. Since these brain areas play crucial roles in both cognitive control and language control [7, 10, 15], these findings suggest that bilingualism leads to neurological changes in control mechanisms. In contrast, some studies did not reveal any difference between bilinguals and monolinguals in certain executive control tasks in behavioral data [4, 16, 17].

Considering the incongruent results reported, it has been noted that bilingualism is not a monolithic construct [12, 18]. Rather, bilingualism is a multifaceted and complex phenomenon, which involves variations in a myriad of factors, such as age, age of acquiring the second language, language proficiency, language context, language experience, language use, linguistic distance [9, 19–22]. Therefore, viewing bilingualism as an all-or-none phenomenon and adopting the binary design of comparing bilinguals with monolinguals may have obscured the potential cognitive effects of heterogeneous characteristics in various bilingual sub-populations, leading to mixed evidence [21, 23].

Indeed, a new trend in examining the bilingual cognitive control focuses on testing whether various bilingual experiences modulate bilingual cognitive control in order to identify the sources that may lead to bilingual effects on cognition and brain plasticity [19–21, 23–27]. For example, DeLuca et al. [25] found that the L2 immersion were related to different patterns of structural plasticity in brain regions associated with cognitive control, such as the right caudate, right putamen, bilateral thalamus. Critically, among a range of variables related to bilingual language use, language switching is a unique language experience that bilingualism offers. It is widely believed that language control is required in proficient bilinguals when switching between two languages frequently in daily life, particularly during language production in contexts where bilinguals choose the target language based on specific interlocutors (i.e., the dual-language context proposed in the Adaptive Control Hypothesis [28]). This particular language use experience could be one of the influential factors of the change in cognitive control mechanisms as a result of the bilingual experience. One reliable window to examine the modulation of bilingual experience on cognitive control mechanisms and brain plasticity is to investigate how training in language processing affects cognitive control [27, 29, 30].

Currently, only a few studies have investigated how language switching training impacts domain-general executive control. Firstly, in one of our previous studies, Zhang et al. [31] trained Chinese-English bilinguals in a language switching task and tested them with an AX version of the Continuous Performance Test (AX-CPT). In this task, letters A and B were cues followed by letters X or Y as probes, and participants were asked to press the YES key only when they saw the AX combination. Results showed that the N2 component, a negative-going component peaking at around 200 ms after stimulus onset, was enlarged at the cue phase after training in the training group, but not in the untrained control group. These findings suggest that language switching training enhances bilinguals’ proactive control abilities, i.e., modulating the activation levels of stimuli prior to their activation [32], specifically strengthening attention control for cue detection. In addition, Timmer, Calabria, and Costa [33] found that two sessions of language switching training led to a larger reduction in switching costs (differences between switch trials and non-switch trials) in a non-linguistic color-shape judgment task, as compared to blocked picture naming training. These results also indicate that language switching training can improve non-linguistic cognitive control. To the best of our knowledge, no previous studies have examined the functional connectivity variations underlying the language switching training effects on domain-general executive control. This type of evidence will further reveal the neuroplasticity of bilinguals induced by the factor of language switching.

In the current study, we aim to further examine how language switching training shapes the neural correlates of cognitive control mechanisms in bilinguals. We are interested in the connections among the critical brain regions involved in both language control and cognitive control. We randomly assigned 46 Chinese-English bilinguals into an experimental group and a control group. At the pre- and post-test sessions, both groups performed a task switching task. During the training phase, the experimental group received language switching training for eight consecutive days. As revealed in our previous study [34], the brain network of language control and domain-general cognitive control involves at least 10 crucial regions, including the bilateral middle frontal gyrus, the dorsal anterior cingulate cortex/ pre-supplementary motor area (dACC/pre-SMA), the left inferior parietal lobule, the bilateral anterior insula/ inferior frontal gyrus (AI/IFG), the left caudate, the bilateral thalamus, and the cerebellum. By analyzing the connectivity patterns among those regions of interest (ROIs) using the extended unified structural equation models (euSEM), we aimed to examine the influence of language switching training on the neural network of cognitive control from the connectivity perspective. Based on previous findings that language switching training can facilitate domain-general cognitive control [33] and that daily language switching experience affects functional connectivity [19, 21, 35, 36], we predicted that language switching training would change the pattern of effective connection of cognitive control network. This attempt will contribute to our understanding of the neurocognitive adaptation to the specific language experience of language switching and provide insights for the current debate on whether bilingualism causes adaptations in the brain.

## Materials and methods

### Participants

Forty-six Chinese-English bilinguals (28 females, M = 22.7 years, SD = 2.1 years) were randomly assigned to experimental and control groups. All were right-handed healthy young adults with normal or corrected to normal vision. None reported a history of neurological or psychiatric diseases. Data from three participants were excluded from further analyses because of excessive head movement (i.e., > 3 mm), data from two participants were excluded due to low accuracy (i.e., < 70%). Also, one participant dropped out of the study after the pre-test session. Therefore, the final sample consisted of 20 participants in each group. They were well matched on age, gender, second language (L2) proficiency measured by the College English Test Band 4 (CET-4), L2 age of acquisition (AOA), and fluid intelligence (see Table 1). CET scores were normalized by average (i.e., 500) and standard deviation (i.e.,70). The full score is 750. For self-rating scores of language proficiency, the between-subject factor Group (experimental group vs. control group) and within-subject factor Language (first language (L1) vs. L2) were examined in a 2-way ANOVA. The self-rating score of L1 proficiency (M = 8, SD = 1.4) was significantly higher than that of L2 proficiency (M = 5.5, SD = 1.3), *F*(1, 38) = 124.62, *p* < 0.001, *η*_*p*_^*2*^ = 0.766. The main effect of group was not significant, *F*(1, 38) = 2.68, *p* = 0.11, *η*_*p*_^*2*^ = 0.066. The interaction between language and group was not significant, *F* < 1. These patterns suggested that participants in both groups were unbalanced bilinguals with a dominant L1. The self-reporting language switching questionnaire was used to measure language switching experience in their daily life on a 5-point scale [37], which quantified the frequency of language switching: never (1), rarely (2), occasionally (3), frequently (4), or always (5). Average level of contextual switching (e.g., There are situations in which I always switch between two languages) for both groups were 1.6 and 1.45, respectively. There was no significant difference between two groups, *t*(38) = 0.536, *p* = 0.595.

**Table 1:** Subject demographics. Means (standard deviations) of the subject demographics for the experimental and control groups.

	Experimental Group	Control Group	*t* value/Chi^2^ value	*p* value
	(*N* = 20)	(*N* = 20)		
Age	22.6 (2.25)	22.7 (2.08)	0.07	0.942
Gender	11 females	13 females	0.42	0.519
CET-4 score	516 (46.66)	533 (61.65)	1.0	0.323
L2 Age of acquisition (AOA)	8.8 (2.43)	8.5 (2.6)	0.37	0.710
Fluid intelligence	56.1 (3.32)	55.3 (4.74)	0.64	0.527

### Procedure

**Fig 1:**
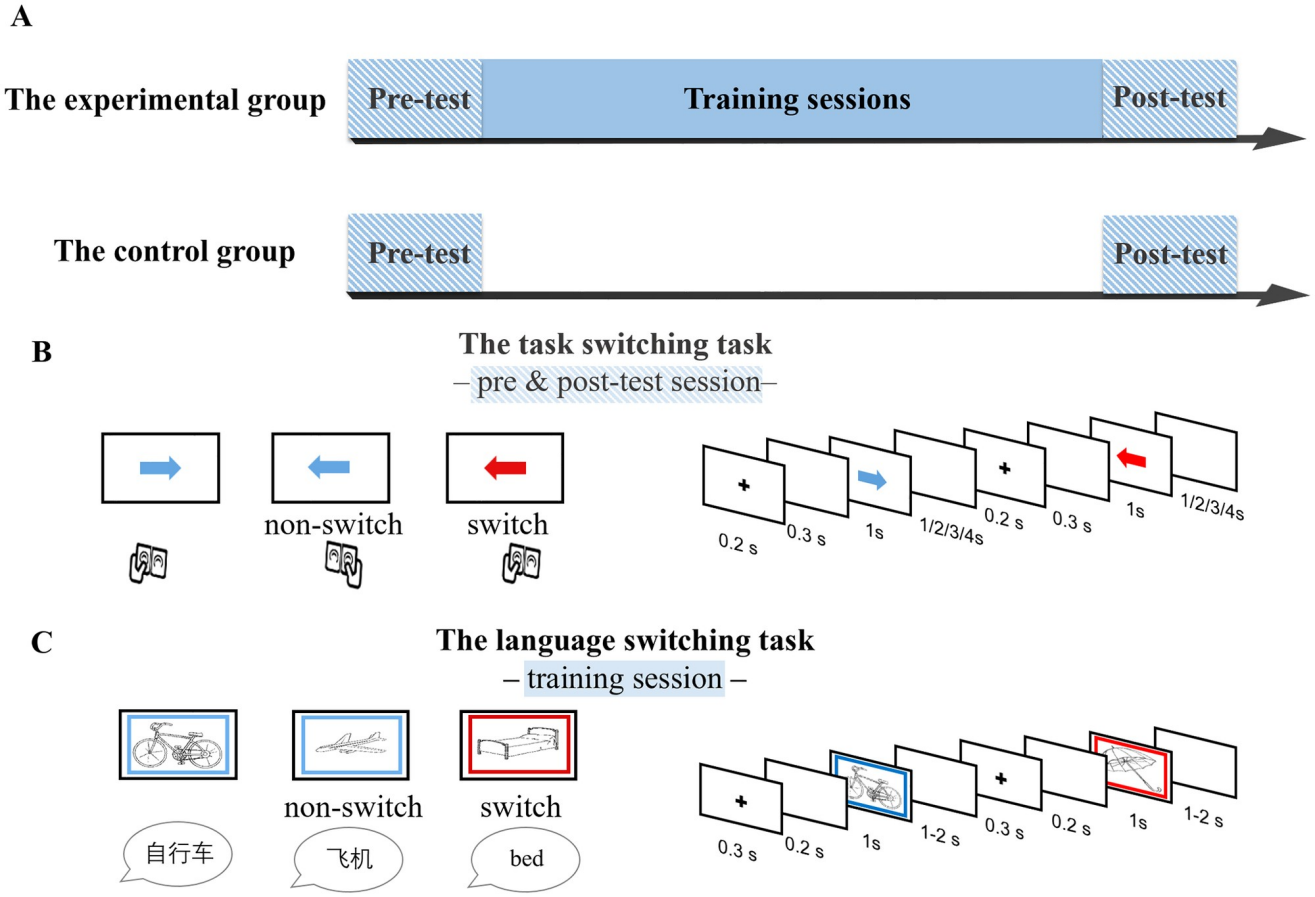
Schematic overview of the experimental design and task. (**A**) Two groups performed the same task switching task at the pre- and post-test fMRI sessions. During the training session, the experimental group received training on a language switching task, whereas the control group did not receive any training. (**B**) Illustration of the task switching task: press the key on the same or opposite side to which the arrow pointed according to the color of arrow. (**C**) Illustration of the language switching task: name the picture in either Chinese or English indicated by the color of the frame.

The study was approved by the Ethical Committee of the State Key Laboratory of Cognitive Neuroscience and Learning at Beijing Normal University. Informed written consent was obtained from all participants. As shown in Fig 1, all participants completed identical pre and post-tests with an interval of 8 days. The experimental group received an 8-day training session between the two test sessions, while the control group did not receive any training.

### Pre- and post-test sessions

#### The task switching task

In the MRI scanner, all participants completed the task switching task. The stimuli were red and blue arrows, either pointing to the right or the left. Participants followed different rules to make a response according to the color of the arrow presented (red or blue). When the arrow was in one color (e.g., red), they were asked to press the key on the same side to which the arrow pointed (e.g., press the key on the left when the arrow pointed to the left). When the arrow was in the other color (e.g., blue), they were supposed to press the key on the opposite side to which the arrow pointed (i.e., press the key on the right when the arrow pointed to the left). On switch trials, the colors of arrows in two consecutive trials were different. In contrast, on non-switch trials, the arrows in two consecutive trials were of the same color. Each trial began with a fixation cross presented for 200 ms, followed by a blank screen of 300 ms. Then, a red or blue arrow, pointing either to the left or to the right, was presented in the center of the screen for 1000 ms. The participants were asked to press the appropriate key with either their left or right thumb as accurately and quickly as possible. The arrow was then replaced by a blank screen jittered randomly for 1, 2, 3, or 4 seconds before the next trial started (i.e., the inter-stimuli intervals). The color-rule assignment was counterbalanced across participants. Half of the trials were switch trials. Participants’ response times and accuracies were automatically collected by the E-Prime 2 software (Psychology Software Tools, Pittsburgh, PA).

Both the pre-test and post-test contained 2 runs, each of which consisted of 82 stimuli and lasted for 5 minutes 28 seconds. On the first day, participants received structural scan after functional scan. Then they completed Raven Standard Progressive Matrices outside MRI scanner. On the last day, all participants filled out the Language History Questionnaire after scanning.

### Training sessions

#### The language switching training task

Participants in the experimental group received language switch training for eight consecutive days. A total number of 48 black-and-white line-drawings were selected from the Snodgrass and Vanderwart database [38]. Eight of them were used as practice trials, and the rest of the pictures were used in the formal experiment. A blue or red frame serving as the language cue was presented simultaneously with a picture inside it. Participants were asked to name the picture in either Chinese or English indicated by the color of the frame as quickly and accurately as possible. A trial began with a blank screen of 300 ms, followed by a fixation cross of 200 ms. Then, a picture with a color frame was presented for a maximum duration of 1 second and disappeared upon a detected naming response. Finally, a blank screen was presented for 1 or 2 seconds before the next trial. Two languages were used to name two successive pictures on switch trials, whereas the same language was used to name two consecutive pictures on non-switch trials. The cue-language mapping was counterbalanced across participants. Participants were trained on the same color-language association during training sessions. Lasting approximately 30 minutes, training on each day included three sessions with four blocks in each session. Each block consisted of 41 trials. On each training day, 40 pictures were presented randomly, and every picture was repeated 6 times for switch and non-switch conditions. Participants’ response times were recorded by an E-prime Serial Response Box (Psychology Software Tools, Pittsburgh, PA) with a plugged-in microphone, while their naming responses were recorded by a digital recorder and transcribed by experimenters to collect naming accuracies.

### fMRI data acquisition

Pre- and post-test sessions were conducted in the 3T Siemens Sonata MRI (Magnetic Resonance Imaging) scanner (Trio Systems, 12-channel sense head coil, TR = 2,000 ms, TE = 20 ms, FOV = 200×200, matrix size = 64×64, 33 axial slices per volume, 164 volumes per each run, in-plane resolution = 3.1mm×3.1mm, slice thickness/gap = 4 mm/0.8 mm). Thirty-three axial slices were collected with an interleaved acquisition order. Each run was preceded by 4 dummy scans that were discarded prior to analyses. After the functional MRI scan, a high-resolution structural MRI scan was acquired for each participant (144-slice T1-weighted image, TR = 2530 ms, TE = 3.93 msec; flip angle = 7°, slice thickness = 1.33mm, resolution within slices = 1.0 × 1.0 mm^2^).

### fMRI image preprocessing

fMRI pre-processing was performed with DPABI [39]. The first 4 volumes from each subject were discarded to allow for magnetization equilibrium. The remaining 160 volumes were corrected for time delay between different slices by resampling with the middle (thirty-third) slice in time as a reference point and realigned to the first volume to estimate the head motion parameters. Data from three participants were excluded due to rotation larger than 3.0 or displacement larger than 3 mm. Next, T1-weighted anatomical images were co-registered to functional images followed by normalization by using EPI templates at a re-sliced voxel size of 3×3×3 mm^3^. Finally, the images were smoothed using a Gaussian kernel with 6×6×6 mm^3^ full-width at half maximum (FWHM).

### Effective connectivity analysis

Firstly, based on our previous study [34], effective connectivity analyses among 10 ROIs was conducted by using extended unified structural equation models (euSEM) via the GIMME program [40]. GIMME generates individual maps and a group map based on connections shared by the majority of individuals (i.e., more than 75%). Criterion setting was based on the probability of detecting whether a true connection should exist in a given sample by sets of simulated data [41] and was used in previous empirical data [42–44]. Two criteria were met in the final model: confirmatory fit index (CFI) values > 0.90, nonnormed fit index (NNFI) values > 0.90. At the post-test session, data from one participant in the control group were removed due to the lack of convergence of the model. Specifically, this participant’s fit index was beyond 3 standard deviation of the group average fit index. Therefore, 20 participants in the experimental group and 19 participants in the control group were entered in the further analyses separately for pre- and post-test sessions.

Furthermore, the nodes of network for each group at the pre-test session were divided into core and periphery regions to detect hubs based on an optimal core-periphery subdivision algorithm [45]. The core-ness (Q) was computed to quantify the goodness of the optimal core-periphery subdivision, with positive values indicating a possible presence of core-periphery structure [46], using brain connectivity toolbox (http://www.brain-connectivity-toolbox.net). The core brain regions were densely connected, while peripheral ones were sparsely connected.

In addition, degree means the number of neighbors a node has, reflecting importance of nodes in the network. In the directed network, a node is important if there are many other nodes that link to it (*k*^*in*^), or if it links to many other nodes (*k*^*out*^). In social networks, in-degree (i.e., a person with many followers) is more important than out-degree (i,e., someone who follows many other people) (Fornito et al., 2016). In brain networks, in-degree and out-degree are also informative. That is, out-degree represents what influence the central nodes exert on other nodes, and in-degree allows us to identify putative sinks of information that receive a large amount of afferent information [47]. As for detected hubs, we calculated the in-degree $k_{i}^{in}$, i.e., the number of edges pointed from other nodes to *i*th node, and out-degree $k_{i}^{out}$, i.e., the number of edges pointed from *i*th node to other nodes, from individual connectivity maps for each group at both test sessions.

Subsequently, to examine the language training effect on the cognitive control network, permutation tests were performed to examine whether the nodal degree showed significant changes between pre- to post-test sessions in both groups. More precisely, half observations were drawn without replacement from original dataset and assigned to one set, all remaining observations were assigned to another set. We could compute difference between two sets of observations by one permutation. Through 1000 permutations, null distribution of random difference was obtained. Then, *p* values of true difference between pre-test and post-test could be calculated according to null distribution. If a *p* valve was smaller than significance threshold (i.e., *p* ≤ 0.05), null hypothesis (i.e., there is no significant difference across test sessions) would be rejected.

### Behavioral results

#### Training sessions

For the experimental group, we examined language switching costs, i.e., difference between switch trials and nonswitch trials over 8 training sessions to see if language switching training would induce any improvement of language control as shown in our previous study [48]. First, reaction times for correct trials below 200 ms or above 1500 ms were excluded from analyses as outliers (absolute outliers: 0.9%). Secondly, we rejected reaction times more than 2.5 standard deviations from the mean of each individual (relative outliers: 2.3%).

**Fig 2:**
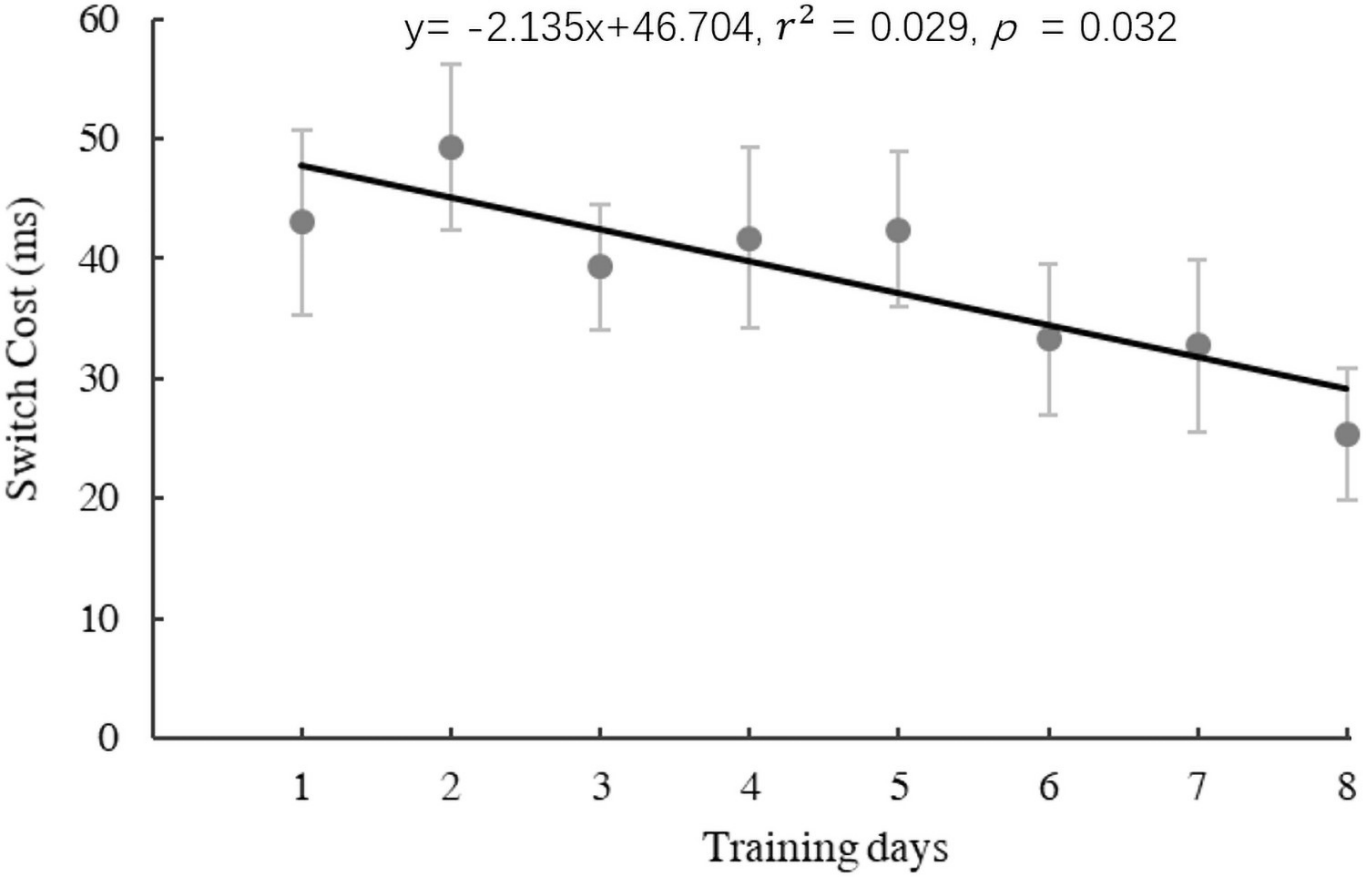
The scatter plot and significant regression line across 8-day training in the experimental group. Switch cost = switch–non-switch, L1 slowing effect = L1 –L2.

Linear regression analysis was then conducted on switch costs across 8 training days. As shown in Fig 2, there was a significant decrease in switch costs with training (regression model: y = −2.135x + 46.703, *F*(1,155) = 4.68, *p* = 0.032, *r*^*2*^ = 0.029).

### Pre-test and post-test sessions

Same trimming process was used for data at the pre- and post-test sessions (absolute outliers: 0.2% at the pre-test and 0.5% at the post-test for the experimental group and 0.1% at the pre-test and 0.6% at the post-test in the control group; relative outliers: 2.4% at the pre-test and 2.4% at the post-test for the experimental group and 2.1% at the pre-test and 2.4% at the post-test in the control group). All behavioral results are shown in Table 2.

**Table 2:** Behavioral performance in the task switching task. Means of reaction times and accuracy (standard deviations in parentheses) for the experimental and control groups at the pre- and post-test sessions.

Reaction time	Pre-test		Post-test	
	Switch	Non-switch	Switch	Non-switch
Experimental group	558 (114)	534 (117)	574 (95)	551 (93)
Control group	587 (79)	561 (84)	594 (80)	568 (90)
**Accuracy**				
Experimental group	95% (7%)	97% (4%)	95% (6%)	96% (6%)
Control group	97% (4%)	98% (2%)	95% (5%)	96% (6%)

For reaction times, a between-subject factor Group (experimental group vs. control group) two, and two within-subject factor Trials Type (switch vs. non-switch) and within-subject factor Test Session (pre-test vs. post-test) were examined in a 3-way ANOVA. Results showed that the main effect of trial type was significant, *F*(1, 38) = 51.09, *p* < 0.001, *η*_*p*_^*2*^ = 0.573, suggesting that participants were significantly slower in switch trials (573 ms) than non-switch trials (547 ms). However, the main effect of test session, *F*(1, 38) = 1.86, *p* = 0.18, *η*_*p*_^*2*^ = 0.047, and the main effect of group, *F* < 1, were not significant. Neither the two-way interactions nor the three-way interaction was significant, *F*s < 1.

The same ANOVA analysis was conducted for accuracy data. Results showed that the main effect of trial type was significant, *F*(1, 38) = 8.01, *p* = 0.007, *η*_*p*_^*2*^ = 0.174. However, the main effect of test session, *F*(1, 38) = 3.57, *p* = 0.066, *η*_*p*_^*2*^ = 0.086, and the main effect of group, *F* < 1, were not significant. Neither the two-way interactions (test session by group: *F*s < 1; trial type by group: *F*(1, 38) = 1.33, *p* = 0.256, *η*_*p*_^*2*^ = 0.034; test session by trial type: *F*(1, 38)s < 1) nor the three-way interaction was significant, *F* < 1.

### Results for effective connectivity analyses

euSEM analyses were conducted for the data of both groups at the pre- and post-test sessions, respectively. The maps had excellent fits to the data for all participants, in experimental group (pre-test: CFI = 0.9860 ± 0.0050, NNFI = 0.9645 ± 0.0128; post-test: CFI = 0.9875 ± 0.0044, NNFI = 0.9645 ± 0.0094) and control group (pre-test: CFI = 0.9850 ± 0.0051, NNFI = 0.9615 ± 0.0109; post-test: CFI = 0.9840 ± 0.0060, NNFI = 0.9605 ± 0.0136).

**Fig 3:**
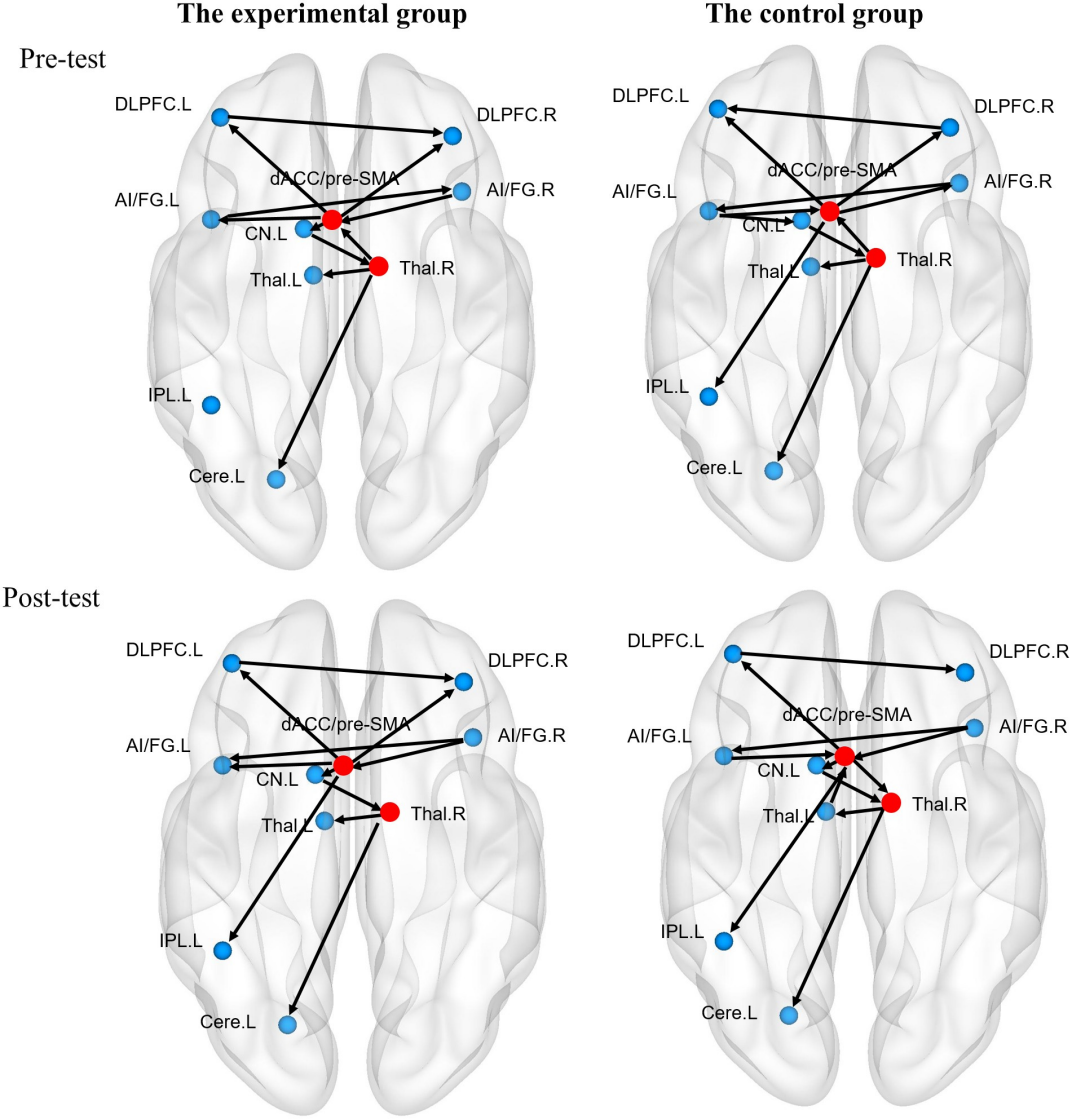
The effective connectivity maps of the experimental and control groups at pre- and post-test sessions. (**A**) The effective connectivity maps of the experimental (left) group and the control group (right) at the pre-test session, (**B**) The effective connectivity maps of the experimental group (left) and the control group (right) at the post-test session. DLPFC. L: the left dorsolateral prefrontal cortex; DLPFC. R: the right dorsolateral prefrontal cortex; dACC/pre-SMA: the dorsal anterior cingulate cortex/pre-supplementary motor area; Thal. L: the left thalamus; Thal. R: the right thalamus; AI/IFG. L: the left anterior insula/inferior frontal gyrus; AI/IFG. R: the right anterior insula/inferior frontal gyrus; CN. L: the left caudate nuclei; IPL. L: the left inferior parietal lobule; Cereb. L: the left cerebellum.

Brain networks were visualized using the BrainNet Viewer [49]. As shown in Fig 3, the dACC/pre-SMA exerted influence on the bilateral middle frontal gyri within the frontal area in both groups at the pre-test session. In addition, the left middle frontal gyrus linked with the right middle frontal gyrus. Besides, information flowed from the dACC/pre-SMA to the left inferior parietal lobule in the control group. Within the subcortical area, both groups showed similar connections from the left caudate to the right thalamus, then to the left thalamus. Additionally, the left insula exerted an influence on the left caudate for the control group. Regarding links between cortical and subcortical areas, dACC/pre-SMA showed feedback loop between the bilateral AI/IFG and received signals from the right thalamus in both groups. The dACC/pre-SMA also connected with the left caudate for the experimental group. Another shared direct connection for both groups was from the right thalamus to cerebellum.

Despite the slight differences based on visual inspection, the hubs of network were shared for both groups, i.e., the dACC/pre-SMA and the right thalamus at the pre-test session (Q = 0.81 for the experimental group; Q = 0.73 for the control group). Subsequently, we examined whether the degrees of hubs exhibited differences between two groups at the pre-test session, and whether degrees of hubs showed different change patterns between both groups from pre-test to post-test session.

At the pre-test, there wasn’t significant different in $k_{i}^{in}$ for experimental and control group ($\Delta k_{dACC/pre-SMA}^{in}=-0.3$, *p* = 0.97; $\Delta k_{right\;thalsmus}^{in}=0.1$, *p* = 0.17). However, $k_{i}^{out}$ in the experimental group is larger than that in the control group ($\Delta k_{dACC/pre-SMA}^{out}=0.7$, *p* < 0.001; $\Delta k_{right\;thalsmus}^{out}=0.5$, *p* = 0.004) at the pre-test session. Thus, we only examined whether the in-degree of hubs would be modulated by training. For the experimental group, $k_{dACC/pre-SMA}^{in}$ at the pre-test session than was larger than that at the post-test ($\Delta k_{dACC/pre-SMA}^{in}=0.7$, *p* < 0.001). In contrast, there was no significant increase in $k_{right\;thalamus}^{in}$ across test sessions for the experimental group ($\Delta k_{right\;thalamus}^{in}=-0.1$, *p* = 0.617). For the control group, there was no significant difference in *k*^*in*^ within the dACC/pre-SMA and the right thalamus across test sessions for control group ($\Delta k_{dACC/pre-SMA}^{in}=-0.7$, *p* = 0.99; $\Delta k_{right\;thalamus}^{in}=-1.0$, *p* = 0.99).

## Discussion

The current study investigated the impact of short-term language switch training on domain-general cognitive control in bilinguals by characterizing the associated changes in functional network connectivity.

First of all, we observed similar connectivity patterns among 10 ROIs crucial to cognitive control in both the experimental and control groups at the pre-test session. Specifically, the dACC/pre-SMA and the right thalamus were identified as hubs within the neural network of domain-general cognitive control for both groups. These two hubs are key components of the cingulo-opercular (CO) network supporting sustained attentional control [50, 51]. Specifically, they transmit information with peripheral nodes to integrate multiple signals both within the CO network and across networks [50]. The dACC is linked to a diversity of cognitive functions, including detecting conflicts, monitoring performance, and exerting control selection [7, 51–53]. Shenhav et al. [54] showed that the dACC evaluated the expected value of control to a given trial and allocated control resources to optimize behavior based on random switching rules. Also, noninvasive brain-stimulation studies using transcranial direct current stimulation (tDCS) and transcranial magnetic stimulation (TMS) techniques have confirmed the causal role of the pre-SMA in inhibitory control [55–59]. For example, Yu and colleagues [59] found that after stimulating pre-SMA using tDCS, participants’ stopping efficiency was enhanced, together with stronger activation in the pre-SMA and increased functional connectivity between the pre-SMA and the ventral medial prefrontal cortex. These results suggest that the interplay between pre-SMA and prefrontal cortex is crucial for cognitive control.

The other hub, the right thalamus has also been shown to be engaged in executive control in various executive tasks, such as the go/no-go task and the N-back task [60]. In our recent study, Wu et al. [34] determined the dACC/pre-SMA as a hub of cortical regions and the right thalamus as a hub of subcortical regions for domain-general cognitive control. In the present study, we again revealed that the dACC/pre-SMA integrated with the bilateral middle frontal gyrus and anterior insula/inferior frontal gyrus, and that the right thalamus served as a hub connecting with the left caudate and the left thalamus during domain-general task switching. Consistent with previous studies, our findings provide additional evidence for the significant roles of the dACC/pre-SMA and the thalamus in domain-general cognitive control.

More importantly, the present study revealed that language switching training significantly reduced the incoming link of the dACC/pre-SMA in the neural network for cognitive control. Specifically, for the experimental group, the dACC/pre-SMA lost connection from the right thalamus during domain-general task switching at the group level after training. In contrast, the control group did not show any significant difference in the hubs’ degrees between pre- and post-test sessions. This connection has been shown to play important roles in both language control and cognitive control [34] and in multiple cognitive tasks [61]. Also, the connection between the posterior ACC and the thalamus has been related to response selection [62]. In the current study, response selection was crucial during both language switching and task switching tasks, as participants needed to select responses for each trial based on randomly presented cues. Critically, previous studies associated lower connectivity with less cognitive costs, suggesting that stronger connectivity is required when executing more demanding cognitive control processes [29, 63, 64]. Thus, the reduction of connectivity from the right thalamus to the dACC/pre-SMA observed in the present study indicates that less neural connectivity is required to complete the same domain-general task and achieve similar behavioral performance after language switching training. This finding is also in line with Zhang et al. [31] ERP results, which indicate that language switching training improves the proactive aspect of domain-general cognitive control, i.e., preparatory goal maintenance before target presentation. More specifically, Zhang and colleagues [31] found that the magnitude of the N2 component in cue-locked ERPs was enlarged after training in the experimental group only. In ERP literature, an enlarged N2 has been linked to more cognitive resources allocated for cognitive control processes, such as cue detection, conflict resolution, and inhibition of irrelevant information, and the neural generator of the N2 component is located in the ACC [65, 66]. Therefore, the increased N2 in the cue phase has been interpreted as evidence suggesting that language training enhanced proactive domain-general cognitive control. Showing reduced connection from the right thalamus to the dACC/pre-SMA induced by training, the current results provided further evidence for the impact of language switching training on domain-general cognitive control. During language switching at the training phase, participants needed to frequently and intensely resolve conflicts from cross-language activation in order to select an appropriate response. Previous studies documented that daily language switching experience shapes functional connectivity related to cognitive control [19, 21, 35, 36]. For instance, bilinguals with more frequent switching experience showed stronger functional connectivity between ACC and bilateral putamen, which was correlated with proactive domain-general cognitive control and was also shown to be critical for language control [24, 67]. Compared with natural language switching context, switching in response to a completely artificial cue in the laboratory is more effortful and requires more intense engagement of language control networks, including the DLPFC and ACC [68]. Training protocol in the present study focused on cued and unpredictable switching process and showed reduced switch costs across eight training days, indicating improvement of language control with intensive exercising of language control [48]. Moreover, such experience with conflict resolution and response selection, especially cued triggered process, involved in language switching changed neural correlated underlying domain-general cognitive control with reduced connectivity from the right thalamus to the dACC/pre-SMA.

One possible underlying reason for such a transfer effect is that shared cognitive processes and common brain networks are involved in both language control and domain-general control [69, 70]. It has been suggested that since bilingual language control and domain-general cognitive control share highly similar brain circuits, these two cognitive processes are highly likely to impact each other [34, 53]. Indeed, many previous studies have shown that bilingual experience shapes both cognitive and neural mechanisms of non-linguistic cognitive control [71–75], implicating that the two types of control have at least some overlap with each other. The current finding offers more direct support for the overlap between language control and domain-general cognitive control from the perspective of training.

As discussed in Introduction, inconsistent results have been obtained in previous studies on bilingual effects on executive functions by comparing monolinguals and bilinguals with various measures such as behavioral performance, electrophysiological responses and neural activation patterns (see [76] for a recent review). It has been proposed that one possible reason for the mixed evidence reported was the dichotomous categorization of bilinguals vs. monolinguals, without the consideration of the heterogeneous profiles of the bilinguals under investigation [21, 23]. Recently, growing research interests have been drawn on investigating the potentially different roles of various bilingual language experiences on cognition and brain plasticity. The training protocol provides a valuable means to determine the potential causal roles of those aspects in modulating cognition and brain [29, 77]. Focusing on the aspect of language switching experience, the current training study provided direct evidence that bilingual language switching experience reduces the neural costs required when performing domain-general cognitive tasks. It has been proposed that language switching, particularly cued language switching, poses extra demands on language control and domain-general control, such as maintaining goals, monitoring conflicts, detecting cues, disengaging from a previous language, engaging a new language [28, 68, 78]. Our results indicate that intensively meeting such control demands during language switching training leads to improved domain-general control abilities. Thus, the current data provide novel evidence for the effect of language switching on cognition and brain plasticity from the perspective of effective brain connectivity. This finding also extends the statement that sustained bilingual experience confers neural adaptations towards increased efficiency [26, 27] by demonstrating the effect of shorter-term language switching training.

Our behavioral results did not reveal significant transfer effect from language-switching training to domain-general cognitive control. We speculate that this could be attributed to the long response stimulus intervals (RSIs) used for the purpose of fMRI scanning. As shown in previous studies [79, 80], longer RSIs cause the sequential congruency effect reflecting conflict control adaption to reduce or even disappear in behavioral performance. As the RSIs in the present study were relatively long (range = 1s to 4s), it is probable that the long RSIs allowed the participants to recover from the influence exerted by the previous trial in the behavioral data. Another possibility for the divergence between our behavioral data and fMRI data and is that the latter is be more sensitive. Such discrepancies have been documented in previous studies testing bilingual effects [9]. Thus, the training effects in our functional connectivity data further suggests that fMRI provides more sensitive measures in detecting the underlying mechanism of bilingual cognitive control than behavioral indices (see [81] for similar discussions). We recognize that one limitation of the current study is that the euSEM analysis cannot generate two independent networks for switch and non-switch conditions. Therefore, we couldn’t examine any specific effect of language switching training on different conditions in the domain-general cognitive control.

In conclusion, our results demonstrated that short-term language switching training modulated the functional organization of non-linguistic cognitive control network. The cross-domain neural plasticity was predominately exhibited in the reduced connection from the right thalamus to the dACC/pre-SMA. The functional reorganization of the cognitive control network provides direct evidence for the contribution of short-term language switching experience in shaping non-linguistic cognitive control. It will be interesting to further investigate how bilinguals’ prior language experience such as daily switching experience and L2 age of acquisition will function with the network reorganization in the future studies.
